# Microstructure and mechanical behavior of metallic glass fiber-reinforced Al alloy matrix composites

**DOI:** 10.1038/srep24384

**Published:** 2016-04-12

**Authors:** Z. Wang, K. Georgarakis, K. S. Nakayama, Y. Li, A. A. Tsarkov, G. Xie, D. Dudina, D. V. Louzguine-Luzgin, A. R. Yavari

**Affiliations:** 1 WPI Advanced Institute for Materials Research, Tohoku University, Sendai 980-8577, Japan; 2 Euronano SIMaP, Institut Polytechnique (INP) de Grenoble, St-Martin-d’Hères, 38402, France; 3 Institute for Materials Research, Tohoku University, Sendai 980-8577, Japan; 4 National University of Science and Technology “MISiS”, Moscow, 119049, Russia; 5 Lavrentyev Institute of Hydrodynamics, Siberian Branch of the Russian Academy of Sciences, Novosibirsk 630090, Russia; 6 Novosibirsk State University, Novosibirsk, 630090, Russia

## Abstract

Metallic glass-reinforced metal matrix composites are an emerging class of composite materials. The metallic nature and the high mechanical strength of the reinforcing phase offers unique possibilities for improving the engineering performance of composites. Understanding the structure at the amorphous/crystalline interfaces and the deformation behavior of these composites is of vital importance for their further development and potential application. In the present work, Zr-based metallic glass fibers have been introduced in Al7075 alloy (Al-Zn-Mg-Cu) matrices using spark plasma sintering (SPS) producing composites with low porosity. The addition of metallic glass reinforcements in the Al-based matrix significantly improves the mechanical behavior of the composites in compression. High-resolution TEM observations at the interface reveal the formation of a thin interdiffusion layer able to provide good bonding between the reinforcing phase and the Al-based matrix. The deformation behavior of the composites was studied, indicating that local plastic deformation occurred in the matrix near the glassy reinforcements followed by the initiation and propagation of cracks mainly through the matrix. The reinforcing phase is seen to inhibit the plastic deformation and retard the crack propagation. The findings offer new insights into the mechanical behavior of metal matrix composites reinforced with metallic glasses.

Al-based metal matrix composites (MMCs) belong to a technologically attractive class of materials with low density, high specific strength and high wear resistance^1^. Improved mechanical properties can be achieved by combining the properties of ductile metallic matrices with the high strength of reinforcements, such as SiC, Al_2_O_3_ or carbon nanotubes. In the past two decades, metallic glasses have been widely studied and are considered as promising candidates for the next-generation structural materials due to their high strength, large elastic limit and high corrosion resistance^2^. Metallic glasses have been suggested as an alternative reinforcement for metal matrix composites that can significantly enhance the mechanical strength while retaining desirable plastic strain before fracture^3^,^4^,^5^,^6^,^7^,^8^,^9^,^10^,^11^,^12^,^13^,^14^,^15^,^16^,^17^. The metallic nature of the reinforcing phase plays an important role in achieving good bonding between the matrix and the glassy reinforcement and overcoming many of the usual shortcomings of the conventional ceramic reinforcing phases, such as interfacial reactions, agglomeration of particles and porosity^5^,^18^. However, up to date, the amorphous/crystalline interfaces in MMCs have not been well studied at the atomic scale. Such studies would be of great fundamental interest and vital importance for advanced structural applications. The nature of the interface plays a significant role determining the mechanical behavior and deformation mechanisms of composite materials. For example, poor adhesion, pores or the formation of brittle intermetallic phases at the interface between matrix and reinforcements may result in premature and catastrophic failure of the composites^1^.

Although metallic glass-reinforced MMCs are still in their early development stage, the progress in the field has been recently reviewed by Dudina *et al*.^19^ and Jayalakshmi *et al*.^20^. The main limitation of the metallic glass-reinforced composites lies in the metastability of the glassy reinforcement and potential crystallization during sintering. Several methods have been used to fabricate such composites. Quite often metallic glass-reinforced composites are fabricated by hot pressing^10^,^11^,^12^,^13^,^14^,^15^,^16^, whereas infiltration casting has been also used in some cases^5^.

A promising consolidation technique for temperature-sensitive materials is spark plasma sintering (SPS), which is based on the simultaneous application of dc pulses and pressure to the powder compact. SPS is a well-developed rapid sintering technique with great potential for efficient consolidation of fine-grained crystalline materials^21^,^22^,^23^,^24^ and producing bulk specimens from amorphous alloy powders within short sintering times suppressing or limiting crystallization events^25^,^26^,^27^. Parts of various sizes or shapes can be successfully produced by SPS. Applying SPS to consolidation of metal-metallic glass composites has been proven efficient in terms of preservation of the amorphous phases^28^,^29^. However, the consolidation behavior of the composite powders during SPS depends on a set of parameters that, among others, include particle morphology, strength and applied pressure. The electric current passing directly through the sample may cause local melting of the material due to overheating of the inter-particle contacts. Overheating of contacts between composite particles (agglomerates) can lead to undesirable chemical reactions and microstructural non-uniformities^30^,^31^. Local overheating can be avoided if a sufficiently high pressure is applied to reduce the resistance of the inter-particle contacts. For a given composite material and powder production method, proper SPS parameters should be selected to avoid these issues, and for that, experimental research is needed.

In the present work, we use the SPS technique to fabricate metallic glass fiber-reinforced metal matrix composites composed of an Al-Zn-Mg-Cu alloy (Al7075) matrix and a dispersion of Zr-based gas atomized metallic glass fibers (15 vol.%) that include nano-wires, micro-wires and micron size particles. The resulting microstructures and the mechanical behavior in compression have been analyzed.

## Results

**Figure 1:**
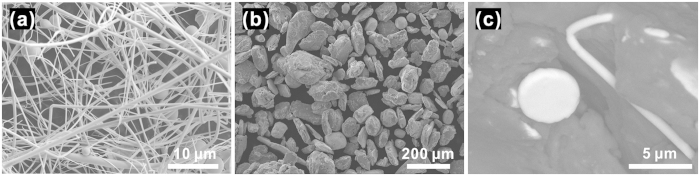
SEM micrographs. (**a**) As-received Zr-based metallic glass fibers fabricated by gas atomization. (**b**) Mechanically milled mixture of Al7075 powders and glass fibers and (**c**) mechanically milled mixture of Al7075 powders and glass fibers in higher magnification (bright areas correspond to the glassy phase, dark areas correspond to the Al7075 alloy).

**Figure 2:**
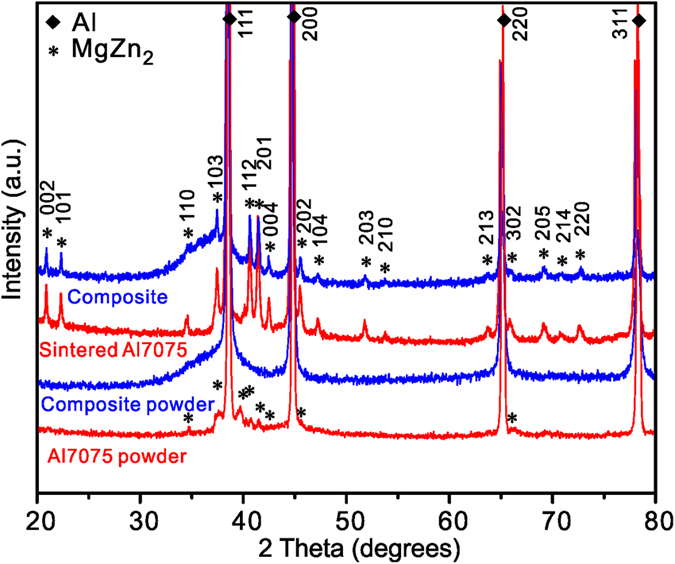
X-ray diffraction patterns. The as-received Al7075 powder, the mechanically milled mixture of powders (85 vol.% Al7075 and 15 vol.% Zr based glassy fiber reinforcements), the spark plasma sintered composite, and the sintered Al7075 alloy without glassy reinforcements.

Figure 1a shows the morphology of Zr-based metallic glass fibers with an average diameter of about 500 nm; wires with larger diameters and quasi-spherical particles with sizes up to 60 μm are also present. The morphology of the powder produced by milling of the Al7075 alloy and the glassy phase is shown in Fig. 1b. The milling process produced a rather homogeneous mixture of powders (Fig. 1b), breaking down most of the long glassy fibers into shorter ones and creating composite particles, in which short glassy fibers were embedded in the Al7075 alloy matrix (Fig. 1c). The composite particles had various shapes from quasi-spherical to platelet-like as a result of repeated welding and fracturing during milling. X-ray diffraction (XRD) patterns of the as-received Al7075 alloy powder, the composite powder, the spark plasma sintered composite (Al7075 + Zr-MG fibers) and the sintered Al7075 alloy without reinforcement are presented in Fig. 2. The as-received (gas atomized) Al7075 alloy powder consists of a fcc-Al solid solution (the major phase) and a minor fraction of η-MgZn_2_ and/or η′-MgZn_2_ precipitates. The XRD pattern of the composite powder shows Bragg peaks corresponding to a fcc-Al solid solution and a broad diffraction maximum corresponding to the amorphous phase, suggesting that no crystallization of the glassy fibers occurred during milling. The peaks corresponding to the MgZn_2_ phase could not be discerned in the XRD pattern of the composite powder suggesting that the precipitates dissolved into the Al-based matrix during milling. The XRD pattern of the spark plasma sintered composite shows Bragg peaks of a fcc-Al solid solution and a broad diffraction maximum indicating that the glassy fibers and particles retained their amorphous structure during sintering. In addition, (smaller) Bragg peaks indicate the precipitation of the η-MgZn_2_ phase (crystal system: hexagonal, space group: P63/mmc) during sintering.

**Figure 3:**
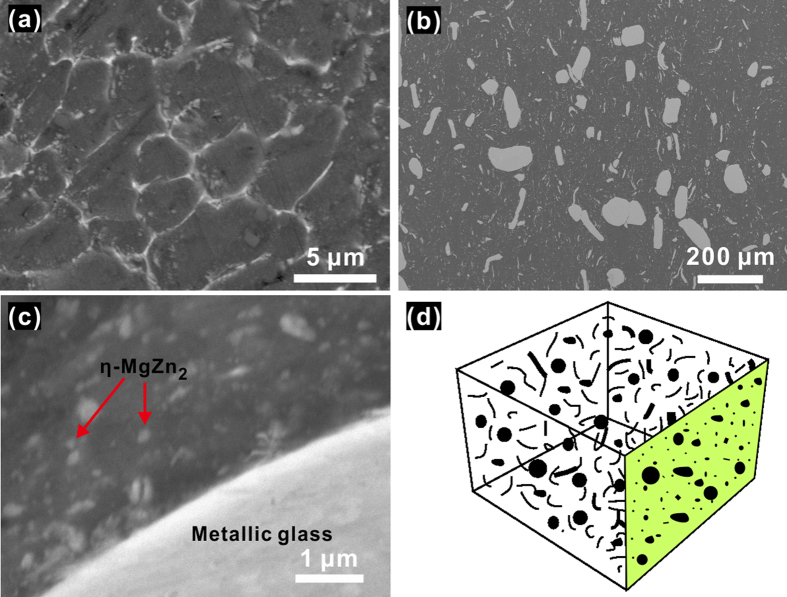
Microstructure of the spark plasma sintered Al7075 alloy and composite. (**a**) Sintered Al7075 specimen without glassy reinforcements. (**b**,**c**) Sintered composite (Al7075 + glassy fibers). (**d**) Schematic showing the random orientation of the glass fibers in the Al7075 matrix.

Figure 3a shows the microstructure of the sintered Al7075 alloy consolidated without any reinforcement additions, whereas Fig. 3b,c show the microstructure of the spark plasma sintered composite. In the sintered Al7075 alloy (Fig. 3a), the secondary phase precipitates at the grain boundaries. EDX microanalysis and mapping (not shown here) indicate that the precipitated phase is rich in Mg and Zn. The XRD analysis, Fig. 2, suggests that the phase precipitating in the Al solid solution matrix is the (hexagonal) η-MgZn_2_ phase. The precipitation of the stable η-phase (MgZn_2_) can be attributed to heat treatment that took place during sintering at a temperature of 573 K, which is higher than the temperatures often used for aging of Al7075 alloys (400–480 K). Aging leads to the formation of coherent Guinier-Preston (GP) zones and the metastable semi-coherent η′-MgZn_2_ phase. Thus, the incoherent equilibrium η-MgZn_2_ phase precipitates as a result of the following sequence: supersaturated solution → GP zones → η′-MgZn_2_ → η-MgZn_2_^32^.

Microstructures shown in Fig. 3b,c (cross-section of the sintered composite) indicate efficient densification achieved by the SPS. The bright areas in Fig. 3b,c correspond to the Zr-based metallic glass fibers and particulates. The glassy phase is rather homogeneously distributed in the Al7075 alloy matrix (dark areas). The interface between the glassy phase and the Al7075 matrix is free of pores and products of interfacial reactions. Although the number of the coarse fibers (with diameters higher than 1 μm) is small compared to the nano fibers, their area fraction is relatively large, as can be seen in Fig. 3b. It is not easy to clearly observe the shape of the glassy fibers in the cross-sectional images in Fig. 3b,c; due to a random distribution of the glassy fibers (schematically shown in Fig. 3d), their cross-sections appear in a variety of shapes. In addition to the glassy phase, the hexagonal η-MgZn_2_ (Fig. 2) secondary phase precipitates in the Al solid solution matrix. Contrary to the case of the sintered Al7075 alloy without reinforcement (Fig. 3a), in the composites (Fig. 3b,c), MgZn_2_ does not precipitate along the grain boundaries, but it is rather homogeneously distributed in the matrix. The average size of these precipitates is of the order of 50 nm.

**Figure 4:**
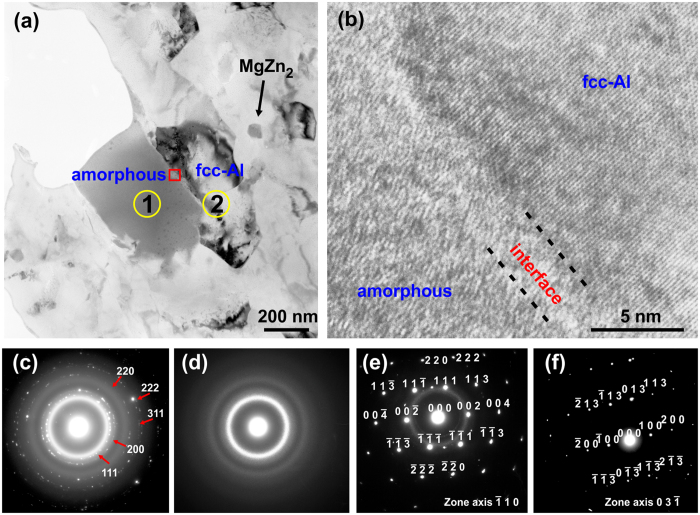
TEM microstructure of the spark plasma sintered composite. (**a**) Bright–field TEM image. (**b**) HRTEM image of the interface between the metallic glass and the fcc-Al matrix. (**c**) SADF of the area corresponding to (**a**) indicating nanocrsytalline fcc-Al and amorphous structure. (**d**) SADF of the area 1 in (**a**) indicating amorphous structure. (**e**) SADF of the area 2 in (**a**) indicating fcc-Al structure. (**f**) SADF of the precipitate phase indicating the η-MgZn_2_ structure.

The microstructure of the composite was further investigated by high-resolution transmission electron microscopy (HRTEM) and selected area electron diffraction (SAED), as shown in Fig. 4. The constituent phases, which are the fcc-Al matrix, amorphous reinforcement and precipitates in the alloy matrix, can be clearly seen in Fig. 4a. The SAED pattern of the area corresponding to Fig. 4a shows a broad ring and several “spotted rings”, which indicate an amorphous phase and a nanocrystalline fcc-Al, respectively. Furthermore, SAED nano-diffraction patterns taken from area 1, area 2 and the MgZn_2_ precipitate of Fig. 4a are shown in Fig. 4d–f, respectively. The pattern in Fig. 4d confirms the fully amorphous structure of the metallic reinforcement. The SAED in Fig. 4e shows a fcc-Al structure pattern together with a diffuse ring of weak intensity corresponding to an amorphous phase. The diffuse ring is presented in the pattern because the selected area is close to the metallic glass inclusion. The precipitate was confirmed to be the η-MgZn_2_ phase (Fig. 4f) which is in accordance with the XRD patterns (Fig. 2). The crystal lattice of η-MgZn_2_ is hexagonal with lattice constants: a = b = 0.518 nm, c = 0.852 nm, Alpha = Beta = 90°, Gamma = 120°. The size of the fcc-Al grain is in the range of 0.2–1 μm and the size of η-MgZn_2_ precipitate is ~50 nm, as estimated from the TEM images.

The interface between the metallic glass and the fcc-Al matrix was observed using HRTEM (Fig. 4b). A thin interphase layer of the order of 2–3 nm is formed at the interface as a result of a short to medium range atomic diffusion. This thin inter-diffusion layer can provide good bonding between the matrix and the reinforcing phase enabling efficient load transfer from the matrix to the reinforcements upon mechanical loading. The interface was observed to be free from pores and other defects. In addition, the TEM-SAED observations (Fig. 4) suggest that the formation of brittle intermetallic phases at the interface, which could potentially lead to brittle failure^33^, was avoided. The limited diffusion between the two phases favors interfacial bonding and is due to fast consolidation of the powders at a moderate temperature (573 K).

**Figure 5:**
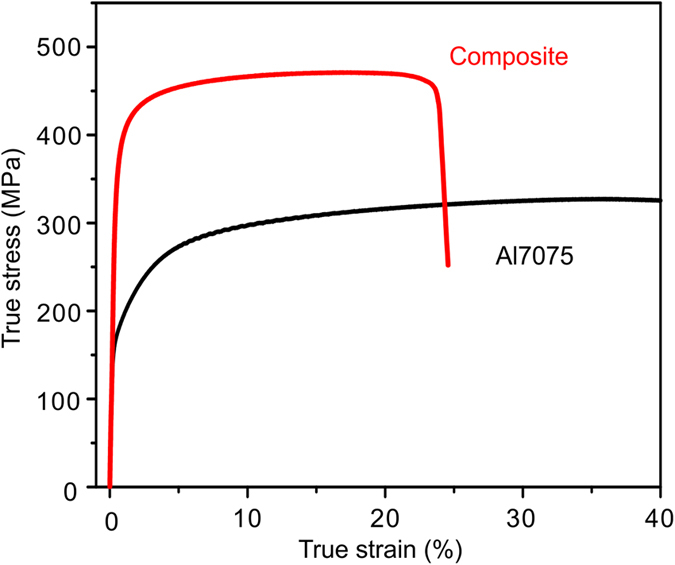
Mechanical properties of spark plasma sintered materials. Stress-strain curves of the composite (15 vol.% Zr based glassy fiber reinforcements in an Al7075 matrix) and the sintered Al7075 alloy for comparison.

Figure 5 shows a typical room temperature uniaxial compression stress-strain curve for the metallic glass fiber-reinforced Al7075 matrix composite together with a curve for the unreinforced sintered Al7075 alloy. The yield strength *σ*_*y*_ (366 ± 6 MPa) of the composite is more than twice higher than that of the sintered Al7075 matrix alloy (168 ± 5 MPa). Similarly, the ultimate compressive strength increases from 326 ± 4 MPa for the sintered Al7075 alloy to 471 ± 7 MPa for the composite. The composite retains a plastic strain of about ~25% before failure.

**Figure 6:**
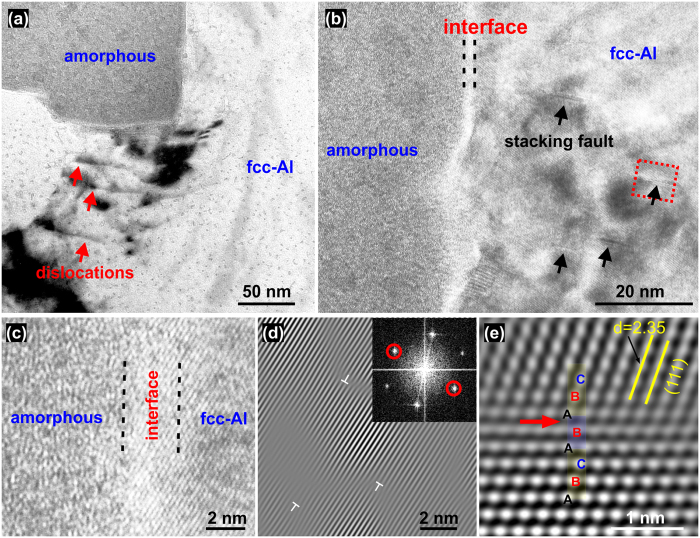
TEM observations of the composite near the interface between the glassy reinforcement and fcc-Al matrix after deformation. (**a**) TEM micrograph indicating the existence of dislocations near the interface. (**b**) TEM micrograph showing stacking faults near the interface. (**c**) High-resolution TEM micrograph showing a close-up of the interface between the matrix and the glassy reinforcement. (**d**) Reconstructed TEM image from the inverse fast Fourier transform (IFFT) using the reflections indicated by red circles in the inset indicating dislocations in an area of the Al matrix close to the interface; the inset corresponds to the fast Fourier transform (FFT) pattern. (**e**) Reconstructed IFFT-TEM image of the area highlighted by red dash rectangular in (**b**) showing the stacking faults at the atomic scale.

The sample after compressive deformation of ~25% was studied by HRTEM. Figure 6 shows TEM images taken from a cross section of the deformed sample. A high density of stacking faults (SFs) (Fig. 6b) and dislocation pile-ups (Fig. 6a) was observed in the fcc-Al grains near the reinforcement/matrix interface. Figure 6c, shows the interface at a higher magnification. The matrix-reinforcement interface in the deformed sample did not show any signs of decohesion between the phases. Furthermore, dislocations can be clearly observed in the TEM image of Fig. 6d which corresponds to the inverse fast Fourier transform (IFFT) image of an area in the Al matrix close to the interface. Figure 6e shows the inverse fast Fourier transform image of the stacking fault region (marked by red dash rectangular in Fig. 6b), which clearly shows a stacking fault pointed by the red arrow.

## Discussion

The enhanced mechanical properties of the metallic glass fiber-reinforced Al7075 matrix composite, Fig. 5, can be rationalized by considering several strengthening mechanisms^16^,^34^. It should be noted that for such a composite, precise determination of their relative contributions is a non-trivial task. The increase in the yield strength from 168 MPa for the Al 7075 unreinforced sintered alloy to 366 MPa for the composite reinforced with the metallic glass fibers is close to the upper bound, ~383 MPa, calculated by the rule of mixtures using Voigt’s Model^35^:


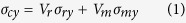


where *V* is the volume fraction, *σ*_*y*_ is the yield strength and the subscripts *c*, *r* and *m* indicate the composite, the reinforcement and the matrix, respectively. The yield strength of the glassy phase used in the calculation is 1600 MPa^36^. The Young’s modulus of the glassy phase is 78 GPa^36^ and that of the matrix is ~70 GPa. The Young’s modulus of the composite calculated by the rule of mixtures (equation 1) is ~71 GPa, which is in good agreement with the experimental value of ~73 GPa.

One of the strengthening mechanisms operating in the composite is the Orowan strengthening, according to which an increased stress is needed for the dislocation motion within the matrix with the fibers and particles serving as obstacles to this motion. In the composite materials examined in this work, two kinds of particles would enable this strengthening mechanism: firstly, the short glass fibers and particles with a wide distribution of sizes and inter-particle distances, and secondly, the nanometric (~50 nm) η-MgZn_2_ phase precipitates, which are rather homogeneously dispersed in the Al solid solution matrix (precipitation hardening). The reinforcing phases cause dislocation pinning and arrest in the matrix resulting in the formation of dislocation pile-ups and stacking faults, as observed near the metallic glass particles in Fig. 6. The value of the effect can be calculated by^37^:


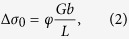


where *φ* is a constant of the order of 2, *G* is the shear modulus and *b* is the Burgers vector. *L* is the distance between the reinforcing particles which can be estimated as:


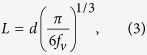


where *d* is the diameter of the particles and *f*_*v*_ is the volume fraction. The Orowan strengthening becomes significant for particles smaller than 1 μm^37^; therefore, only the glass fibers, whose diameter is smaller than 1 μm were taken into account. The contribution of the Orowan strengthening was estimated to be of the order of ~90 MPa due to the nano-sized glassy fibers and the secondary precipitates.

The second strengthening mechanism operating in composites is the dislocation strengthening of the matrix due to the generation of dislocations during the preparation process, also known as Taylor strengthening. In the composites investigated in this work, dislocations are generated during the ball milling process of the matrix together with the reinforcing phase, where the initially soft Al 7075 particles become heavily deformed. In addition, dislocations may be generated during the consolidation process due to the thermal expansion mismatch between the matrix and the metallic glass reinforcement. However, the relative contribution of this effect will be less pronounced than in the case of MMCs reinforced with ceramic particles due to a much greater thermal expansion mismatch in the latter. This contribution can be calculated by the Taylor formula^38^,^39^:


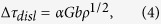


where *α* is a constant of the order of 0.5–1 and *ρ* is the dislocation density given by the following formula:


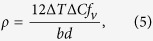


where Δ*C* is the difference in thermal expansion coefficients between the matrix and the reinforcing particles, Δ*T* is the temperature change from the processing temperature to room temperature. In this study, *α* is taken as 1 and the linear thermal expansion coefficient of the Zr-based metallic glass is equal to 11.3 × 10^−6^ K^−1 ^40. The shear strength Δ*τ*_*disl*_ was calculated to be about 25 MPa, which gives a dislocation strengthening due to the thermal expansion mismatch of the order of 75 MPa, according to σ ≈ 3.1*τ*^39^.

A third strengthening mechanism is the grain refinement of the matrix (Hall-Petch strengthening effect) as a result of the ball milling process of the Al7075 powders together with the glassy reinforcement. TEM observations revealed that the grain size of the matrix is of the order of 0.2–1 μm. Sintering at 573 K for only 10 min does not allow extensive grain growth and thus the grains of the Al-based matrix of the composite remain smaller than the (micron- sized) grains of the sintered Al7075 (Fig. 3a). In addition to these micro-mechanisms that contribute to the strengthening of the composite indirectly through the microstructure of the matrix, direct strengthening can result from the load transfer from the matrix to the reinforcing fibers and particles, shifting mechanical yielding of the matrix phase to higher stresses. In this work, the total strengthening of the composite is of the order of 200 MPa compared to the sintered matrix alloy (Fig. 5) and about 270 MPa compared to a fully annealed matrix alloy (Al7075-O); therefore, the strengthening due to grain refinement and direct strengthening is estimated to be in range of 30–100 MPa.

**Figure 7:**
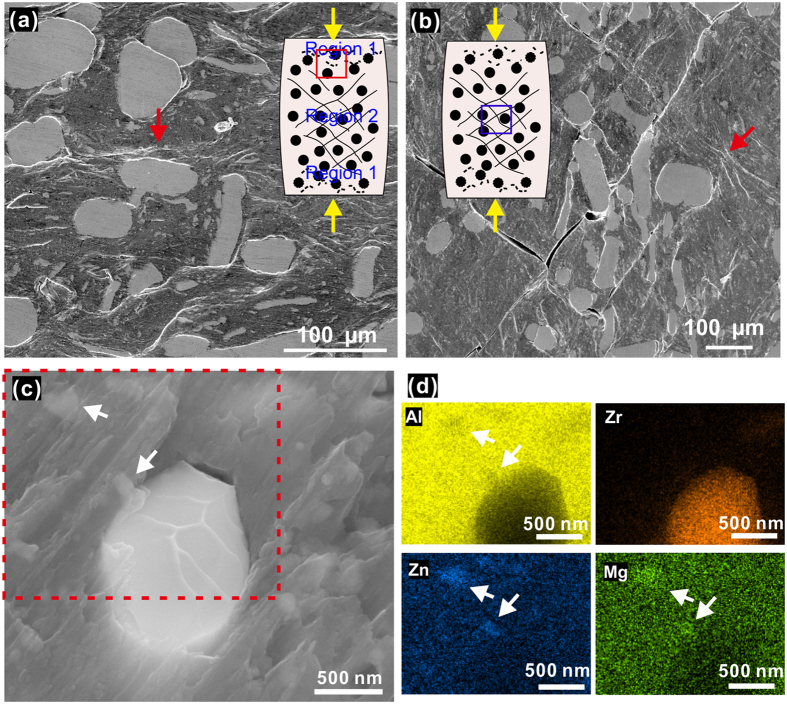
(**a,b**) SEM micrograph of the lateral surface of the deformed composite material (after fracture), the inserted schematic diagrams show the morphology of the composite during compression, the black circles represent large glassy particles, the white matrix represents the Al7075 + nanoscale glassy fibers, the black curves represent the cracks which are along the maximum shear stress direction, the black dash lines represent the deformation bands due to plastic deformation in the matrix. (**c**) SEM micrograph of the fracture surface of the composite after failure. (**d**) SEM-EDX mapping of area marked by a red rectangle in (**c**), which indicates that the bright area is glassy reinforcement (rich in Zr element and poor in Al, Mg and Zn), the spots marked by white arrows are MgZn_2_ precipitates (rich in Mg and Zn).

Understanding the deformation processes and crack formation during loading is important for the design of the composites. In order to further investigate the deformation behavior of the composite, the morphology of the lateral surfaces (parallel to the loading axis) of compressed samples has been examined by SEM, Fig. 7a,b. Typical micrographs were taken from different regions of a deformed composite sample, as schematically indicated in the insets of Fig. 7a,b. Region 1 (Fig. 7a) represents the areas near the top and bottom surfaces in contact with the crossheads, whereas region 2 represents the central area of the sample, where the stress and strain during compression reach their maxima (Supplementary information). The regions close to the bottom and top surfaces (Fig. 7a) show many zones of heterogeneous deformation that manifest the plastic deformation in the matrix. Region 2 (Fig. 7b) exhibits more cracks formed along the planes of the maximum shear stress at approximately 45° with the loading axis. In addition, zones of heterogeneous deformation in the form of localized deformation bands can also be seen in Region 2, red arrow in Fig. 7b, as a result of the plastic deformation in the matrix prior to the formation of cracks. These deformation bands, which are the result of the slip band operation and accumulation in the Al alloy matrix, are mesoscopic features of the plastic deformation and can be considered as potential precursors for the formation of cracks. The direction of the localized deformation bands (red arrow in Fig. 7a) in region 1 is on average normal to the loading axis, whereas in region 2 their direction (red arrow in Fig. 7b) forms an angle of about 45° with the loading axis and is parallel to the maximum shear stress direction. The deformation of the matrix is significantly affected by the presence of the glassy reinforcements, as indicated by the change in the direction of the localized deformation bands. The bands are seen to bend around the nano/micro-sized metallic glass reinforcements indicating how the reinforcements impede the plastic deformation in the matrix. The density of the deformation bands is higher in areas close to the metallic glass reinforcements acting as stress concentrators. Furthermore, the glassy reinforcements act as obstacles to cracks causing crack deflection and/or blunting and thus retarding their propagation. It is interesting to note that no decohesion of the glassy phase from the matrix can be observed after the compression tests, implying good bonding between the glassy phase and the Al alloy matrix. This is evidenced by the SEM observations on the fracture surface (Fig. 7c) and high resolution TEM studies of deformed composite specimens (Fig. 6b,c), during which no debonding events were detected between the glassy fibers and the matrix. Interestingly, a typical vein-like pattern of the fracture of metallic glasses appears on the fracture surface of the micron-sized glassy fiber of Fig. 7c. In the same figure, nanometric MgZn_2_ precipitates can be observed embedded in the matrix, also confirmed by the elemental analysis maps of Fig. 7d.

**Figure 8:**
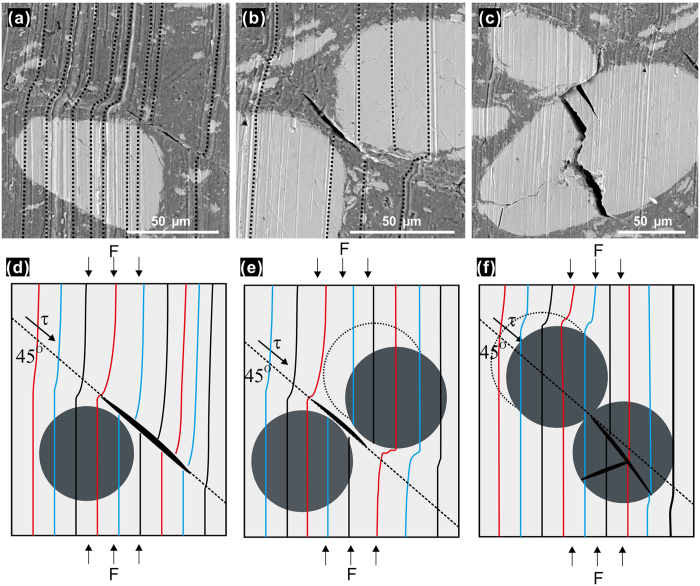
(**a–c**) The lateral surface of the compressed sample after plastic deformation of 6% (the parallel scratches were added intentionally along the loading direction before the test, marked with black dashed lines to guide the eye). (**d**–**f**) The schematic diagrams showing the origination of cracks during compressive deformation, the distorted colored lines are to guide the eye on the shear deformation near the reinforcements (the lines were straight before deformation), the grey circles represent large particles of glassy reinforcement and the dashed circles indicate their position before deformation, the black area represents the cracks, *τ* is the shear stress and F is the loading force.

In order to understand better the deformation behavior of the composites, parallel grinding traces were intentionally added on the lateral surfaces of a sample before deformation along the loading direction, marked as black dashed lines in Figs 8a and 6c. The images were taken from an area in between Region 1 and Region 2 as indicated in the inset of Fig. 7a. After ~6% of plastic deformation, the direction of the parallel traces (black dash lines) is severely distorted near large glassy particles and in the direction of the maximum shear stress planes, indicating higher local strains near the reinforcements. In addition, minor cracks are formed in the vicinity of the larger glassy particles due to the shear deformation in the matrix (Fig. 8a,b). In very rare cases, cracks may also occur in the glassy particles; such behavior was observed only in cases where two glassy particles touched each other during deformation, Fig. 8c. Noteworthy, the cracks created in the glassy particles do not propagate into the matrix, which is due to the plasticity of the matrix and crack blunting and arrest occurring near the interface. In Fig. 8d–f the sketches depict the deformation behavior and the formation of cracks, as observed in Fig. 8a–c, respectively. Shear deformation tends to occur in the matrix near the glassy particles mainly because of the large difference in the yield stress between the glassy phase and matrix. In addition, the reinforcing fibers and particles act as stress concentration sites leading to preferential local micro-deformation of the matrix and creation of voids and/or microcracks near the reinforcing phase. The maximum shear stresses at about 45° degrees with the loading direction lead to the coalescence of voids, which eventually causes the nucleation and propagation of cracks.

The overall deformation, crack formation and propagation mechanisms observed in metallic glass reinforced composites may differ from those typically observed in conventional metal matrix composites with ceramic reinforcements. This difference is due to the metallic nature and the unusual properties of the glassy reinforcing phases. A sufficiently strong bonding at the interface is achieved mainly due to short (and medium) range atomic diffusion between the reinforcement and the matrix, resulting in a thin (2–3 nm, Figs 4b and 6) inter-diffusion layer at the interface. Although many metallic glasses show limited ductility in tension, their toughness is much higher (one order of magnitude or greater) than that of brittle ceramic reinforcements^41^. Therefore, upon loading and deformation, the glassy reinforcement is able to bear loads without cracking or fracture of the metallic glass fibers in most of the cases (the cracking of the glassy phase shown in Fig. 8c is an exceptional case, which does not represent a typical behavior). Cracks initiate and propagate along the zones of localized deformation within the matrix and the reinforcing phase is able to inhibit the plastic deformation and retard crack propagation. In addition, in fiber-reinforced MMCs, the difference between the Poisson’s ratio of the matrix and that of the fiber reinforcement lead to radial stresses imposed on both. These radial stresses can result in radial tensile stresses at the interface and cause debonding between the reinforcement and the matrix. However, the difference between the Poisson’s ratios of the Zr-based metallic glass (Poisson’s ratio is 0.35–0.37)^42^ and Al7075 alloy (Poisson’s ratio is ~0.33) is small compared to metal-ceramic pairs. As a result, this radial tensile stress at the interface is small, but is inevitable when the composite is subjected to external stresses. It should be noted that sufficient bonding strength between the glassy particles and the matrix is necessary for sustaining severe local plastic deformation and avoiding premature debonding and failure at the interface. As seen in Fig. 8a,b, cracks originated in the matrix close to the glass reinforcement and propagated through the matrix rather than along the interface, indicating good bonding between the glassy particles and the matrix.

The strengthening achieved by the introduction of metallic glass in an Al7075 matrix shows that spark plasma sintering is a promising method to synthesize light metal matrix composites with metallic glass reinforcements. The noticeable plastic strain (>20%) retained before fracture indicates the potential for further increases in mechanical strength of the composite materials by increasing the volume fraction of the amorphous reinforcements and by exploring the precipitation hardening mechanism through thermomechanical treatment and aging processes. The good bonding between the metallic glass and the Al-based matrix is a major advantage of these composites since poor adhesion between matrix and reinforcement is often a cause of premature failure degrading the mechanical properties of composites and their reliability.

In summary, Al-alloy matrix composites containing Zr-based metallic glass fiber reinforcements were fabricated by spark plasma sintering (SPS). Due to a moderate temperature and short sintering time, the glassy fibers retained their amorphous structure throughout the consolidation process. The metallic glass fibers were distributed homogenously in the matrix and had good bonding with the Al7075 alloy matrix. The inter-diffusion layer between the metallic glass fibers and the Al-alloy matrix was found to be about 2–3 nm thick. The composites with 15 vol.% of the glassy reinforcements exhibited a significant increase in the yield strength (from ~168 MPa for Al7075 alloy to ~366 MPa for the composite). At the same time, the composites showed about 25% of plastic strain before fracture. The introduction of metallic glass fiber reinforcements in Al-based matrices can significantly increase the mechanical strength of the composites through various strengthening mechanisms. Local plastic deformation and crack initiation was observed near the glassy reinforcements, whereas failure at the interface was avoided due to the good bonding between the reinforcements and the matrix. In addition, the glassy reinforcements were observed to impede crack propagation in the matrix by crack blunting and arrest, thus contributing to the plasticity retained before fracture.

## Methods

Zr_65_Cu_18_Ni_7_Al_10_ (at. %) metallic glass fibers (Zr-MG fibers) produced by gas atomization^43^ were selected as the reinforcing phase (Fig. 1a). The product of gas atomization consisted mainly of nano- and micro-wires but also contained micron-sized particles. The reinforcing phase was mixed with gas atomized Al7075 alloy powder (5.79% Zn, 2.45% Mg, 1.74% Cu, 0.08% Fe, 0.05% Si, remainder Al; wt%) having an average particle size of ~150 μm using a Retsch PM100 planetary ball mill with hardened steel balls and vials. The milling time was 2 h and a rotational speed of 150 rpm was used. The fractions of the Zr-MG fibers and Al7075 powder were 15 vol.% and 85 vol.% respectively. A few drops of ethanol were added into the milling vials as a process control agent. The composite powders were pre-compacted and then sintered by spark plasma sintering (SPS). Sintering was carried out in vacuum using a SPS system (Model SPS-3.20MK-IV), at a heating rate of 50 K/min (from room temperature to 543 K) and 5 K/min (from 543 K to 573 K), where 573 K was the sintering temperature. A pressure of 600 MPa was applied during the whole sintering process including the heating step. The sintering time was 10 minutes. The sintered specimens had a cylindrical shape with 15 mm diameter of and 5 mm height. In addition, gas atomized Al7075 powders without any additives were sintered by SPS using the same processing parameters for comparison. The microstructure of the composites was studied using a scanning electron microscope (SEM, JEOL, JSM-7800F) equipped with a field emission gun and an energy dispersive X-ray spectrometer (EDX). Transmission electron microscopy (TEM) observations were carried out using a JEM-2010F microscope (JEOL Co. Ltd.) with an aberration coefficient of objective lens of 1.0 mm operated at 200 kV. The phase composition of the powders and sintered composites was studied by X-ray diffraction (XRD) with Cu K_α_ radiation. Uniaxial compression tests were performed at room temperature at a loading rate of 5 × 10^−4^ s^−1^ using an Instron 5581 testing machine. Rectangular specimens with dimensions of 3 × 3 × 6 mm^3^ and an aspect ratio of 2:1 were cut and polished to a perfect geometry with parallel top and bottom ends, and at least seven samples were tested for the composite and the Al7075 matrix alloy, respectively.

## Additional Information

**How to cite this article**: Wang, Z. *et al*. Microstructure and mechanical behavior of metallic glass fiber-reinforced Al alloy matrix composites. *Sci. Rep*. **6**, 24384; doi: 10.1038/srep24384 (2016).

## Supplementary Material

Supplementary Information
