# Supplementary material for: Microstructure and mechanical behavior of metallic glass fiber-reinforced Al alloy matrix composites
Source: Sci Rep. 2016 Apr 12;6:24384. doi: 10.1038/srep24384 (PMC4828712; doi:10.1038/srep24384)
Supplement: Supplementary Information [file srep24384-s1.pdf]

Supporting Information:

Microstructure and mechanical behavior of metallic glass fiber-reinforced Al alloy matrix composites

Z. Wang[1*], K. Georgarakis[1,2*],
K. S. Nakayama[1], Y. Li[3*], A.A. Tsarkov[4], G. Xie[3], D. Dudina[5,6],
D.V. Louzguine-Luzgin[1] and A.R. Yavari[1,2]

[1]WPI Advanced Institute for Materials Research, Tohoku University, Sendai 980-8577, Japan
[2]Euronano SIMaP, Institut Polytechnique (INP) de Grenoble, St-Martin-d'Hères, 38402, France
[3]Institute for Materials Research, Tohoku University, Sendai 980-8577, Japan
[4]National University of Science and Technology "MISiS", Moscow, 119049, Russia
[5] Lavrentyev Institute of Hydrodynamics, Siberian Branch of the Russian Academy of Sciences, Novosibirsk 630090, Russia
[6] Novosibirsk State University, Novosibirsk, 630090, Russia

The strain evolution of a ductile material in compression was simulated using finite element modeling. The simulation was performed on Al7075 matrix alloy using the DEFORM 2D/3D software. Cylindrical specimens with an aspect ratio of 2 was selected and subjected to compression deformation at 293 K with a strain rate of $5*10^{-4}s^{-1}$. The friction coefficient between contact interfaces of the specimen and dies was selected to be 0.08. The true stress-strain data for Al7075, obtained from the uniaxial compression experimental study (Fig. 5), is used as input data for the simulation.

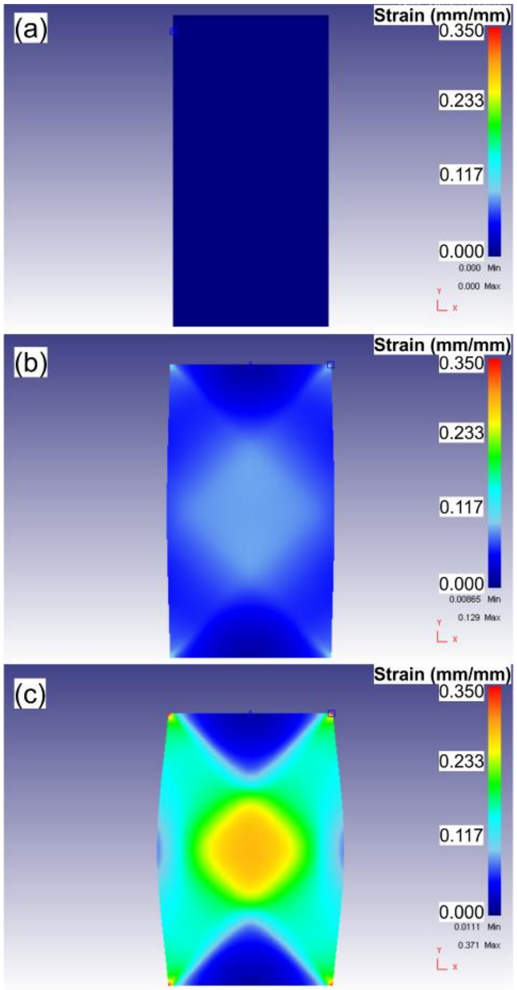

Figure S1. Finite element modeling of compressive deformation of the Al7075 alloy at room temperature. (a) at 0% deformation, (b) after 6.5 % deformation, (c) after 13 % deformation.

Fig. S1 shows the strain distribution in the specimen at different compression stages. The undeformed specimen is shown in Fig. S1a. Figures S1b and S1c show the distribution of strain in the specimen after 6.5 % and 13 % deformation respectively. The strain is not homogeneously distributed to the whole volume, but fields with higher strain appear at the center of the specimen. Favorable directions of strain appear at about 45 degrees to the loading axis, along the maximum shear stress direction. These results are in agreement with the experimental SEM observations on the lateral surfaces of deformed samples (Fig. 7) which reveal higher density of cracks appearing in the center of the specimen with the majority of cracks and deformation zones (deformation bands) formed at an angle of 45 degrees to the loading axis.
